# Impact of Environmental and Climate Factors on Spatial Distribution of Cutaneous Leishmaniasis in Northeastern Iran: Utilizing Remote Sensing

**DOI:** 10.18502/jad.v14i1.2704

**Published:** 2020-03-31

**Authors:** Mohammad Reza Shirzadi, Mohammad Javanbakht, Hassan Vatandoost, Nahid Jesri, Abedin Saghafipour, Reza Fouladi-Fard, Alireza Omidi-Oskouei

**Affiliations:** 1 Communicable Diseases Management Center, Ministry of Health and Medical Education, Tehran, Iran; 2 Department of Remote Sensing and GIS, Faculty of Geography, Tehran University, Tehran, Iran; 3 Department of Medical Entomology and Vector Control, School of Public Health, Tehran University of Medical Sciences, Tehran, Iran; 4 Department of Chemical Pollutants and Pesticides, Institute for Environmental Research, Tehran University of Medical Sciences, Tehran, Iran; 5 Remote Sensing and GIS Centre, Shahid Beheshti University, Tehran, Iran; 6 Department of Public Health, Faculty of Health, Qom University of Medical Sciences, Qom, Iran; 7 Research Center for Environmental Pollutants, Qom University of Medical Sciences, Qom, Iran

**Keywords:** Cutaneous leishmaniasis, Remote sensing, Climate, Iran

## Abstract

**Background:**

Cutaneous leishmaniasis (CL) is a dermal and parasitic disease.. The aim of this study was to determine the effect of environmental and climate factors on spatial distribution of CL in northeastern Iran by utilizing remote sensing from 20 March 2016 to 19 March 2017.

**Methods:**

In this ecological study, the data were divided into two parts: The descriptive data on human CL cases were gathered from Communicable Diseases center of Iran. The remote sensing techniques and satellite imagery data (TRMM, MODIS-Aqua, MODIS-Terra and AMSR-2 with spatial resolution 0.25°, 0.05°, 5600m and 10km) of environmental and climate factors were used to determine the spatial pattern changes of cutaneous leishmaniasis incidence.

**Results:**

The incidence of CL in North Khorasan, Razavi Khorasan, and South Khorasan was 35.80 per 100,000 people (309/863092), 34.14 per 100,000 people (2197/6,434,501) and 7.67 per 100,000 people (59/768,898), respectively. The incidence of CL had the highest correlation with soil moisture and evapotranspiration. Moreover, the incidence of disease was significantly correlated with Normalized Difference Vegetation Index (NDVI) and air humidity while it had the lowest correlation with rainfall. Furthermore, the CL incidence had an indirect correlation relation with the air temperature meaning that with an increase in the temperature, the incidence of disease decreased.

**Conclusion:**

As such, the incidence of disease was also higher in the northern regions; most areas of North Khorasan and northern regions of Razavi Khorasan; where the rainfall, vegetation, specific humidity, evapotranspiration, and soil moisture was higher than the southern areas.

## Introduction

Cutaneous leishmaniasis (CL) is considered a dermal and protozoan infectious disease which is caused by some species of *Leishmania* parasite (1). The disease is more prevalent in many underdeveloped and developing countries (2). Cutaneous leishmaniasis is classified into two forms in terms of clinical symptoms and epidemiological characteristics: Zoonotic Cutaneous Leishmaniasis (ZCL) or Cutaneous Leishmaniasis due to *Leishmania major* (CLM) and Anthroponotic Cutaneous Leishmaniasis (ACL) or Cutaneous Leishmaniasis due to *Leishmania tropica* (CLT). The vectors of CL are some species of female sand flies belonging to the genus *Phlebotomus* such as *Ph. papatasi* and *Ph. sergenti*. Besides, reservoirs of disease are some wild rodents, domestic dogs and humans in ZCL and ACL, respectively (3). The globally recognized foci of the disease are located approximately between 28–42 degrees of north latitude (4). Cutaneous leishmaniasis is an endemic disease in tropical areas of the United States, Africa, the Indian subcontinent, and in the subtropical regions of southwest Asia (5). According to the WHO reports, CL has local transmission in 98 countries and over 350 million people are globally at risk (5, 6). More than 20,000 cases of CL are reported annually in Iran, but the actual cases are estimated to be 4 to 5 times more than that figure (7, 8). Iran’s geographic and climatic conditions are suitable for the growth of sand flies as vectors of ZCL, and rodents as reservoirs of disease (3, 9). This zoonotic disease is more prevalent in plain regions in central areas of Iran; such as Qom province (10) and in the old texture of the towns (11, 12). Nowadays, ZCL has endemic foci in 17 provinces of 31 provinces in Iran, such as the three studied provinces (North Khorasan, Khorasan Razavi and South Khorasan) (13–15). Investigating the correlation between climatic factors and incidence of the arthropod born diseases as applied epidemiologic studies can help epidemiologists in prevention and disease control, ultimately leading to taking preventive measures in vulnerable areas (16). In this regard, Salmon et al. (2012) studied the climate change and the prevalence of leishmaniasis in Argentina. The findings of this study indicated that the prevalence of this disease has increased with nonlinear climate changes (17). Chamaillé et al. (2010) revealed that the disease prevalence is high in two regions; one region with low winter temperatures, 1042mm average annual rainfall and much forest cover and second area with higher temperatures, lower rainfall and less forest cover. They declared two region groups probably responsible to the environment factors favored by the two sand fly vectors species; *Ph. ariasi* and *Ph. perniciosus* respectively (18). In addition, Gonzalez et al. studied the relationship between climate change and risk of leishmaniasis in North America. The results of the study showed that climate changes and increase in temperature provide a natural environment and suitable breeding places for living vectors of this disease (19). In 2012, Tommy et al. conducted a standard multiple regression study to investigate the effect of climate factors on ZCL incidence in central Tanzania and showed that in case of rising rainfall as 1mm and a 1% increase in humidity from July to September, lead to an increase in incidence of the disease (20). Ghatee et al. in a study on role of environmental and climatic risk factors on the occurrence of cutaneous leishmaniasis, found that some environmental and climatic factors including land cover, slope, elevation, close proximity to cattle sheds, rainfall, and temperature were the most effective factors (21). In addition, the Iranian medical entomologists also have studied on impact of environmental and climate factors on spatial distribution of CL using remote sensing (22, 23). Following the launch of the first meteorological satellites in the 1960s, and Landsat-1 in 1972, the use of remote sensing in human health studies have increased for more than four decades. The information received from satellites and their various sensors with the ability to separate temporal and spatial distribution of data has created a new field in the scientific researches on human health. Several ecological indicators have been used in remote sensing related to vector borne diseases, including Normalized Difference Vegetation Index (NDVI), near surface air and sea temperatures, soil moisture and cold cloudy periods. The term ‘remote sensing’ (RS) is a technique for diagnosis of objects from afar (24). So, this study aimed for determining the impact of environmental and climatic factors on spatial distribution of CL in Northeastern Iran from March 2016 to March 2017 utilizing remote sensing.

## Materials and Methods

### Study area

**Fig. 1.**
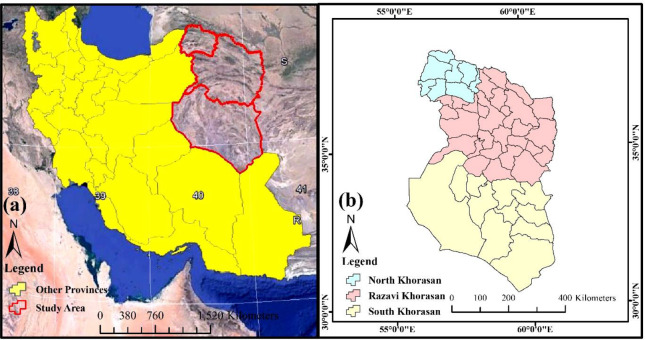
Location of the study area, northeastern Iran

One of the main and confirmed foci of CL in Iran is the northeastern areas of the country (8). Therefore, the three provinces of North Khorasan (55°53′–58°20′ E and, 36° 37′−38°17′ N), Razavi Khorasan (35° 60′ N–59° 15′ E and, 36° 35′ N–60° 25′ E) and South Khorasan (57° 46′–60° 57′ E and 30° 35′−34° 14′ N) were selected as the study area of this research (Fig. 1). According to the general census of population and housing in 2017, the totally residents in the study area was 8,066,491 people (North Khorasan, Razavi Khorasan and South Khorasan provinces had a population of 863092, 6434501 and 768898, respectively). These three provinces have an area of 28311, 118854, and 148669km^2^ , respectively (North Khorasan, Razavi Khorasan and South Khorasan provinces).

### Study design

The data used in this study included two parts: the field data related to the incidence of disease and remote sensing data in a one-year period (from 20 March 2016 to 19 March 2017). Estimation of various climatic components in large spatial scales has always been a frequent problem due to lack of availability of appropriate data. In recent years, a global database based on satellite estimates has reduced these problems. In this research, various products of the Modus sensor and the Global Land Data Assimilation System (GLDAS) were used. The GLDAS aims to produce hydrological and meteorological parameters using the combination of ground-level models and satellite imagery with the aim of studying the changes in the energy and water bill on the ground. Satellite imagery data (TRMM, MODIS–Aqua, MODIS–Terra and AMSR-2 with spatial resolution 0.25°, 0.05°, 5600m and 10km) of environmental and climate factors were used to determine the spatial pattern changes of cutaneous leishmaniasis incidence. This system was developed jointly by the National Aeronautics and Space Administration (NASA), the Goddard Space Flight Center (GSFC), the National Oceanic and Atmospheric Administration (NOAA), and the National Centers for Environmental Prediction (NCEP) (25). The parameters extracted from GLDAS included evapotranspiration, near surface temperature, rainfall, soil moisture and air humidity (Table 1). The GLDAS data has been used and evaluated in many scientific studies (26–28). Furthermore, the Normalized Difference Vegetation Index (NDVI) and Modis data were used in order to study variations in vegetation. In addition, mean of climatic and environmental variables and cutaneous leishmaniasis incidence was evaluated (Table 2). Field data on CL disease is routinely collected from urban and rural health centers in these three provinces using standard epidemiological forms and reported to the Center for Disease Control and Prevention of the Ministry of Health and Medical Education of Iran. In order to investigate the spatial changes of the disease incidence, as well as the relationship between climatic and environmental factors and the incidence of CL, one-year data of this disease were taken from the CDC.

**Table 1.** The characteristics of parameters extracted from GLDAS in this study

**Index**	**Source**	**Unit**	**Spatial Resolution**
**Precipitation rate**	TRMM	mm	0.25°
**Land Surface Temperature**	MODIS - Aqua	C	0.05°
**Normalized Difference Vegetation Index**	MODIS - Terra	NDVI	5600m
**Specific humidity**	GLADS Model	Kg kg−1	0.25°
**Evapotranspiration**	GLADS Model	Kg m−2 s−1	0.25°
**Soil Moisture**	AMSR-2	%	10km

**Table 2.** Mean of climatic and environmental variables and cutaneous leishmaniasis incidence in northeastern provinces of Iran from March 2016 to March 2017

**Index**	**North Khorasan**	**Razavi Khorasan**	**South Khorasan**	**Total**
**Precipitation rate (mm)**	21.13	15.75	8.38	15.08
**Land surface temperature (c°)**	30.07	34.96	42.67	35.9
**Normalized Difference Vegetation Index (NDVI)**	0.15	0.12	0.08	0.11
**Specific humidity (Kg kg−1) x1e−3**	4.64	4.12	3.73	4.16
**Evapotranspiration (kg/m2 -y) x1e−7**	70.0	60.01	43.21	57.74
**Soil moisture (%)**	23.51	14.65	5.01	14.36
**CL incidence/100,000**	35.80	34.14	7.67	25.66
**CL cases**	309	2197	59	2565

### Statistical analysis

The data related to CL disease were entered into Excel software and the disease incidence was calculated. Also, Pearson correlation coefficient was used to determine the relationship between climatic and environmental factors and CL. The correlation coefficient is a value between −1.0 and 1.0, positive correlation implies the presence of a direct relationship between two variables in which, when one variable increases as the other increases. As well as, negative correlation means there is a reverse change between the two variables. The zero correlation, however, means that the variables are independent of each other. Correlation is significant at the 0.05 level.

## Results

**Fig. 2.**
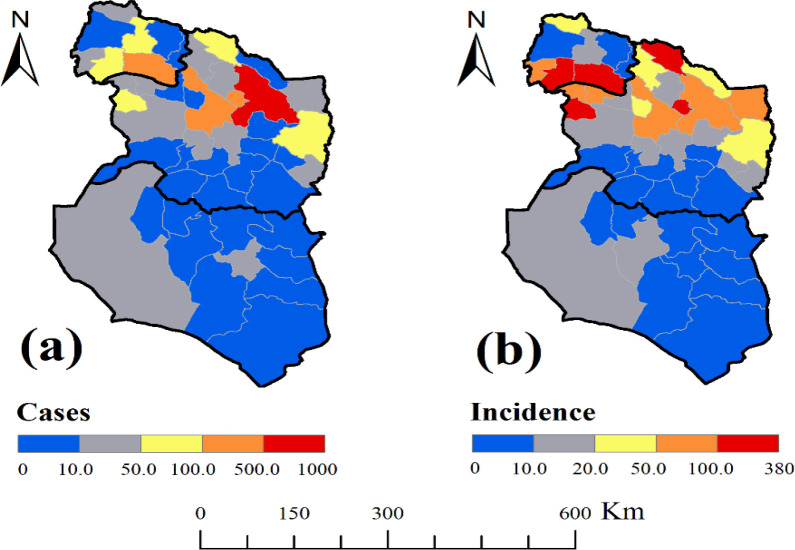
Spatial distribution of cutaneous leishmaniasis in northeastern Iran

The incidence of CL in all three provinces was 25.66 per 100,000 people (2565/8,066,491). Also, CL incidence in North Khorasan, Razavi Khorasan, and South Khorasan was 35.80 per 100,000 people (309/863092), 34.14 per 100,000 people (2197/6,434,501) and 7.67 per 100,000 people (59/768,898), respectively (Table 2 and Fig. 2). Spatial distribution of CL showed that the northern regions had a higher incidence than the southern ones. Therefore, the disease had a north to south trend which tended to increase from south to the north. In terms of changes in the three provinces, the southern regions of North Khorasan and northern regions of Razavi Khorasan provinces had a high incidence. The southern regions of Razavi Khorasan Province and the whole region of South Khorasan had a lower incidence. Table 2 shows the mean of six climatic and environmental variables (rainfall, temperature, vegetation, specific humidity, evapotranspiration and soil moisture) in northeastern provinces of Iran. The relationship between climatic and environmental factors and incidence of CL was evaluated.

### Rain precipitation rate (mm)

**Fig. 3.**
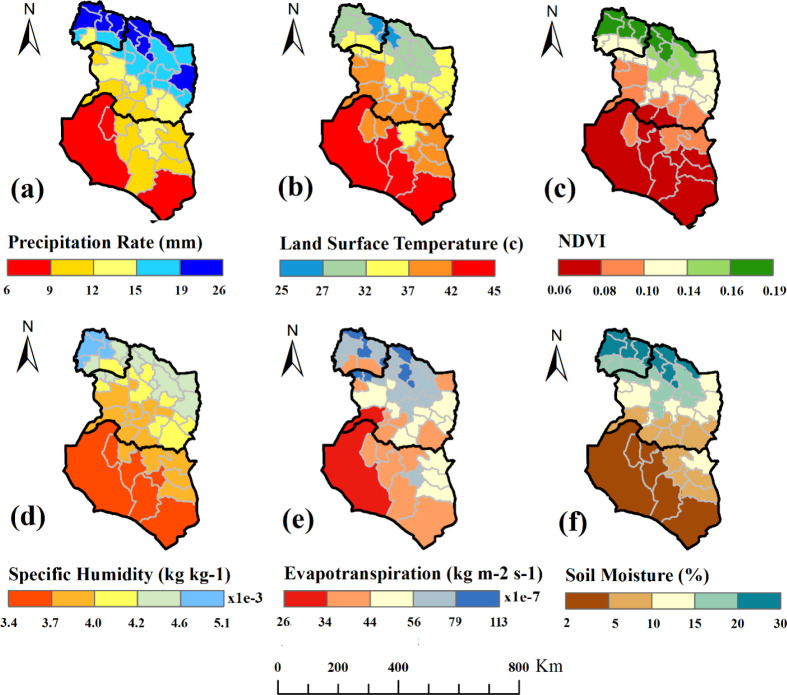
Time averaged map of monthly rate of all environmental and climate factors in northeastern Iran

The results showed that the mean annual precipitation in these three provinces was 15.08mm. The rate of change in average rainfall across these provinces showed a downward trend at a rate of 21.13 to 15.75 and 8.38mm in North Khorasan, Razavi Khorasan and South Khorasan, respectively (Table 2). The average annual rainfall in southern regions; all areas of South Khorasan, central and southern areas of Razavi Khorasan was lower than that in northern regions; most areas of North Khorasan and northern areas of Razavi Khorasan. So that North Khorasan Province had the highest rainfall during the year. The Razavi Khorasan Province also had different conditions; the northern areas of the province had specifically good rainfall, and the southern and western parts had a lower rainfall than the northern parts. Furthermore, South Khorasan Province had a low rate of rainfall in most regions (Fig. 3a). There was a significant correlation between the incidence of CL and the precipitation mean (p= 0.046). According to the figures 2 and 3, it was also observed that the maximum precipitation areas were matched with the maximum incidence of the disease.

### Land surface temperature (°C)

The average temperature in all of these provinces was 35.9 °C. It was 42.67, 34.96 and 30.07° for the three provinces of South Khorasan, Razavi Khorasan, North Khorasan and respectively. In this study, the trend of temperature decreased from south to north areas. In terms of temperature conditions in the three studied provinces, North Khorasan Province had a lower average temperature. The northern and northeastern parts of the Razavi Khorasan Province had lower temperatures than those in the southern and eastern parts of the province. South Khorasan Province residents had also experienced a different temperature in one year, and its southern and western parts had a higher temperature than the eastern and northern regions of the province (Fig. 3b). There was a correlation between incidence of CL and air temperature. It was observed that the incidence of disease had decreased in southern regions with high temperatures.

### Normalized Difference Vegetation Index (NDVI)

Spatial variations in vegetation revealed that the northern regions; most areas of North Khorasan and northern regions of Razavi Khorasan had a better status than the southern regions (Fig. 3c). The NDVI was 0.11 in all studied provinces, while it was 0.8, 0.12 and 0.15 in South Khorasan, Razavi Khorasan and North Khorasan, respectively. The southern and western regions of Razavi Khorasan and all regions of South Khorasan Province had less vegetation. Statistical analysis showed that there was a direct correlation between the trend of vegetation changes and the incidence of disease.

### Specific humidity (SH %)

Spatial variations of specific humidity during this course indicated that the air humidity was low in most regions (Table 2). The highest SH was observed in northwest of North Khorasan Province. The northern parts of Razavi Khorasan Province had higher humidity. In the South Khorasan Province, the southwest areas were less humid (Fig. 3d). In total, the maximum moisture content was consistent with areas having the maximum incidence of disease.

### Evapotranspiration (kg/ m2 −y)

In this study, evapotranspiration had a northwest-southeast trend, showing a decreasing trend from northwest to south east (Table 2). The evapotranspiration of the North Khorasan Province was more than that of the other two provinces. In the province of Razavi Khorasan, the northern and eastern regions of the province had higher evapotranspiration than the southern and western regions. The spatial variations of evapotranspiration in South Khorasan Province showed a low level of this index with most of the regions having low evapotranspiration (Fig. 3e). The comparison of evapotranspiration and spatial changes in the incidence of the disease showed that evapotranspiration was also an effective factor in the incidence of this disease.

### Soil moisture (%)

The spatial variation of soil moisture suggested that the northern regions (most areas of North Khorasan and northern regions of Razavi Khorasan) had better conditions than the southern regions (Table 2). Soil moisture was specifically high in most parts of North Khorasan Province. In Razavi Khorasan Province, the northern and southern regions had higher rates of soil moisture than in the central regions of the province. Moreover, the soil moisture content reached the lowest level in southern Khorasan Province (Fig. 3f). There was also a significantly direct correlation between spatial variation of soil moisture and disease incidence (p= 0.007).

Pearson Correlation Coefficient was used to study the relationship between every environmental and climatic factors and the incidence of the disease. Table 3 shows the Pearson correlation coefficient indices with the incidence of CL.

**Table 3.** Pearson correlation coefficient indices with the incidence of Cutaneous leishmaniasis in northeastern Iran

**Index**	**Correlation coefficient**	**p- value**
**Precipitation rate**	0.239	0.046
**Land surface temperature**	−0.322	0.028
**Normalized Difference Vegetation Index**	0.314	0.032
**Specific humidity**	0.303	0.038
**Evapotranspiration**	0.465	0.001
**Soil moisture**	0.392	0.007

## Discussion

The incidence of CL in North Khorasan, Razavi Khorasan, and South Khorasan was 35.80, 34.14 and 7.67 per 100,000 people, respectively. The epidemiological studies on CL disease in Iran indicated that the majority of cases were reported in eastern, central, and southern provinces in the country (29). In addition, the incidence of CL in Iran were evaluated 27.5 per 100, 000 populations in 2011 (30). So, we can conclude that the CL incidence in two provinces out of 3 studied provinces; North Khorasan, Razavi Khorasan was higher than the average incidence of the country. In the present study, the incidence of CL was highly correlated with soil moisture and evapotranspiration. In addition, there was a moderately positive relationship between vegetation and humidity index with the incidence of the disease. Besides, the incidence of the disease had the lowest correlation with precipitation. In addition, there was also a significantly positive relationship between CL incidence and land surface temperature, so that the incidence of the disease decreased with the increasing temperature. In this study, there was a weak significant positive correlation between the incidence of CL and the mean of precipitation. It was also observed that the maximum precipitation areas were matched with the maximum incidence of the disease. In contrast, Entezari and Eskandari have been found that there was an inverse correlation between climatic parameters of rainfall and specific humidity with CL prevalence in Fars Province, southern Iran (31). It has already been proven that as rainfall increases at the right level, it can usually adjust air temperature and increases humidity. But, flood-causing rainfalls destroy the breeding places of sand flies in addition to reducing their populations, leading consequently to a decrease in the incidence of the disease (32, 33). For example, the *Ph. argentipes* larvae, when exposed to monsoon floods in northern India, ascends the mud walls, and descend after the deposition of water and the normalization of the conditions, but in other species, these floods lead to the death of the sand flies (34). In a study in French Guyana, it was observed that the precipitation had a weak relationship with the CL incidence (35). In another study in Brazil, a direct correlation was found between incidence of disease and precipitation (36). In the present study, there was an inverse correlation between CL incidence and land surface temperature. It has been observed that the incidence of disease has decreased in southern regions with high temperatures. This finding is in contrast with the basic concepts about the disease. It had been previously proved that the growth of all species of sand flies occur at temperatures above 18 °C, which is the threshold temperature for their life (37). In general, the findings of various researchers show that the optimum temperature for the development of their life stages before maturation of sand flies is in range of 18–28 °C (37). In an ecological study on phlebotomine sand flies in the Greek Aegean Islands showed that temperature preferences of them were 21–29 °C (38). It seems in addition to temperature, other environmental and climatic factors are associated to the disease. In addition, adult sand flies activate during sunset and night when the land surface temperature is balanced and humidity rises. In this condition, the infected female phlebotomine sandflies can cause the transmission of *Leishmania* parasites into susceptible hosts such as humans (39). In the present study, spatial distribution of vegetation revealed that the northern regions; most areas of North Khorasan and northern regions of Razavi Khorasan had a better situation than the southern ones. Statistical analysis based on the NDVI showed that there is a direct correlation between vegetation changes and the incidence of disease. Contrary to the results of the present study, Mozafari et al. in a study comparing spatial distribution of disease cases with vegetation status in Ardakan, central Iran, found that CL is more prevalent in areas with less vegetation (40). Such differences in the results of various studies can indicate the impact of different geographic locations on the CL incidence. Sometimes the effects of the same climatic parameters will vary from one region to another. Vegetation is a function of temperature and humidity. The vegetation cover status and the CL incidence are affected by climatic factors (41). Vegetation can be increased under the influence of higher precipitation and lower temperatures. The situation also holds true about CL incidence. In addition, the incidence of CL disease also depends on sand flies’ density (3). Therefore, CL is indirectly affected by climatic factors. The incidence of the disease depends on a variety of factors in addition to the population of sand flies. For example, the change in population and the density of the disease reservoirs (rodents) can affect the incidence of disease (42). At the time of drought, the population of wild rodents approaching human settlements may increase. This situation, in turn, can lead to an increase in the population of rodents infected with *Leishmania* parasite. So, the probability of blood-sucking of sand flies from these rodents rises as well as biting on humans and inoculation of the *Leishmania* parasite to humans (43, 44). Therefore, a vegetation index at a low level may ultimately lead to an increase in disease as well. The average specific humidity in the study area was 32.22%. The highest humidity was observed in North Khorasan and northern regions of Razavi Khorasan. In general, areas with maximum moisture content were consistent with areas with the highest incidence of disease. The specific humidity is favorable for egg hatching, being between 80–90% (45). Hesam-Mohammadi et al. also found that a specific humidity of 24.83–36.33% was desirable for sand flies collected from Kashan district, Central Iran (46). Hence, in the present study area, climatic conditions are favorable for the activity of sand flies in terms of humidity, their biting habit on hosts such as humans and the transmission of CL disease. Although specific humidity is required in breeding places of sand flies, high moisture can make larvae migrate to other places (37). Mozafari et al. analyzed the role of biochemical factors (climatic factors affecting the life of living creatures) and the outbreak of CL in the Yazd-Ardakan plain, central Iran, using data from meteorological data during the statistical period of 1997–2009. Their research showed that maximum disease incidence occurs in the second half of the year, especially in autumn, and there is also a weak positive correlation between specific humidity and incidence of the disease (40). In the present study, evapotranspiration has a northwest-southeast trend which decreases from northwest to south east of all study areas. The comparison of evapotranspiration index with spatial distribution of disease confirmed that evapotranspiration is also an important factor in CL incidence. Water movement in river beds can have an effective role in increasing evapotranspiration and consequently in increasing specific humidity, ultimately leading to temperature modification. As such, North Khorasan with large rivers and streams characterized by sufficient evapotranspiration, has provided suitable breeding places for the activities of sand flies as vectors of the disease. Previous studies have also proved the presence of infected female *P. papatasi* species with *Leishmania major* parasite in endemic areas of North Khorasan Province (13). Spatial changes in the soil moisture indicate that the northern regions have more favorable conditions than the southern ones. In South Khorasan Province, the rate of soil moisture was also the lowest, which associated to the areas with the lowest incidence of the disease. Researchers believe that rotten plants in the soil are the main source of food for sand flies larvae in nature since larvae cannot survive without water and moisture. The findings of the current study support the previous studies evaluating the effect of soil moisture on the trend of the disease. In a study on the mapping of vulnerable areas of Kala-Azar disease using RS and GIS in parts of the Bihar Province of India, it was found that ponds, streams, irrigation canals, and rivers are directly interrelated and also very effective in maintaining soil moisture and subsoil levels with an average of 65–80% for the growth of larva and adult sand flies (47).

## Conclusion

According to the findings, the incidence of disease was also higher in the northern regions; most areas of North Khorasan and northern regions of Razavi Khorasan; where the rainfall, vegetation, specific humidity, evapotranspiration, and soil moisture was higher than the southern studied areas.
